# Bridging care gaps: A qualitative study of postpartum health coaching for hypertensive disorders of pregnancy

**DOI:** 10.1002/pmf2.70354

**Published:** 2026-07-10

**Authors:** Kristine Buchholz, Elizabeth Albert, Megan R. Knutson Sinaise, Srishti Gupta, Melissa Neal, Scott Hetzel, Kara K. Hoppe

**Affiliations:** ^1^ Department of Obstetrics & Gynecology School of Medicine and Public Health University of Wisconsin‐Madison Madison Wisconsin USA; ^2^ Department of Family Medicine and Community Health School of Medicine and Public Health University of Wisconsin‐Madison Madison Wisconsin USA; ^3^ Department of Biostatistics and Medical Informatics University of Wisconsin‐Madison Madison Wisconsin USA

**Keywords:** blood pressure, health coaching, hypertensive disorders of pregnancy, maternal health, postpartum care, qualitative, self‐management

## Abstract

**Introduction:**

Hypertensive disorders of pregnancy (HDPs) affect approximately one in seven US deliveries and are associated with significant adverse maternal health outcomes. Standard postpartum care, typically a single 6‐week visit, is insufficient for ongoing HDP management, education, and support. Combining home blood pressure monitoring with health coaching may enhance self‐management, lifestyle change, and engagement with care, yet patient perspectives remain underexplored. This qualitative study examined 1‐year postpartum experiences among birthing people with HDPs and explored perceptions of a postpartum health coaching intervention.

**Methods:**

This qualitative study was embedded within the STAC‐MyHEARTp randomized trial (NCT05685251), which compared home blood pressure monitoring plus health coaching to usual care. Using an explanatory sequential design, semi‐structured interviews were conducted posttrial and analyzed thematically. Self‐Determination Theory and Self‐Regulation Theory guided coding and interpretation, with comparison across intervention and control arms.

**Results:**

Thirty‐two participants (16 per arm) completed interviews. Four themes were identified: (1) postpartum experiences were emotionally and medically challenging, characterized by fear, anxiety, and hypervigilance; (2) participants strongly desired education and support, with coaching filling gaps unmet by standard care; (3) coaching provided behavioral, emotional, and healthcare benefits, including enhanced motivation, confidence, self‐monitoring, lifestyle change, and self‐advocacy; (4) persistent gaps, particularly in mental health support and continuity of care, remained across groups. Control participants recognized the potential value of structured support.

**Conclusions:**

Health coaching offers meaningful, multidimensional support for individuals with HDP, promoting self‐efficacy, engagement, and lifestyle change. Integrating structured coaching into comprehensive postpartum care can address persistent gaps, improve patient experience, and potentially enhance long‐term cardiovascular and psychosocial outcomes, underscoring the need for sustained, person‐centered postpartum care.

## 1 INTRODUCTION

Hypertensive disorders of pregnancy (HDPs), including chronic hypertension, gestational hypertension, preeclampsia, and eclampsia, affect approximately one in seven deliveries in the United States, disproportionately impacting Black (20.9%), Indigenous (16.4%), rural (15.5%), and low‐income (16.4%) populations [1]. HDPs are linked to adverse outcomes across the perinatal continuum, including placental abruption, preterm birth, low birth weight, Neonatal Intensive Care Unit (NICU) admission, maternal hemorrhage, stroke, and increased maternal and infant mortality [2], as well as long‐term risk of chronic hypertension and cardiovascular disease [3]. These risks highlight the need for ongoing postpartum management, yet routine care—typically a single 6‐week visit—is insufficient. Although authorities recommend long‐term monitoring, transition to primary care, and counseling on modifiable lifestyle factors, these practices are inconsistently implemented, leaving many patients without recommended follow‐up [4].

Home blood pressure monitoring (HBPM) increases postpartum blood pressure ascertainment, improves postpartum visit attendance, and decreases hospital readmissions compared with usual care [5, 6]. National guidelines increasingly recommend combining blood pressure monitoring with supportive interventions, such as structured health coaching or behavior‐change programs, to optimize outcomes [7]. A recent meta‐analysis in nonpregnant adult populations found that health coaching can meaningfully reduce blood pressure, improve dietary behaviors, and enhance self‐efficacy among individuals with hypertension, suggesting it may be an effective and scalable component of hypertension management [8].

Yet evidence on how best to integrate these approaches in pregnant and postpartum populations remains limited, and substantial gaps in implementation and effectiveness persist. Existing studies offer early insight: one proof‐of‐concept trial of remote blood pressure monitoring paired with coaching improved patients’ self‐monitoring and planning skills but did not yield sustained blood‐pressure changes [9], while another feasibility study combining home monitoring with lifestyle support detected high rates of persistent hypertension, highlighting the need for structured postpartum interventions.

Patient perspectives on postpartum hypertension health coaching remain underrepresented in the literature. One mixed‐methods study found that coupling HBPM with medication management was associated with an enhanced sense of control and reduced blood pressure–related anxiety; however, health coaching was not examined [10]. A qualitative study of postpartum individuals with HDPs in Australia found that an intensive coaching model may have increased awareness of cardiovascular risk and motivation to adopt health‐promoting behaviors compared with usual care; however, it remains unclear whether these gains translated into sustained lifestyle changes or greater engagement with healthcare [11]. Developing postpartum hypertension interventions that are effective, relevant, equitable, and responsive to real‐world needs requires centering the voices and experiences of those most impacted.

This qualitative study examined postpartum experiences among birthing people with HDPs and explored perceptions of a 1‐year postpartum health coaching intervention.

## 2 METHODS

### 2.1 Design and theoretical framework

This manuscript presents findings from the qualitative component of the STAC‐MyHEARTp randomized controlled trial (NCT05685251). The trial evaluated a 12‐month intervention integrating self‐monitored blood pressure (SMBP) with telephone‐based health coaching, compared with standard postpartum care, among individuals with HDPs. The trial evaluated a 12‐month intervention integrating SMBP with telephone‐based health coaching, compared with standard postpartum care, among individuals with HDPs. Participants were randomized to intervention (health coaching + SMBP) or control group (usual care) using a computer‐generated randomization table. The intervention was adapted from the original MyHeart trial intervention [12] and modified for use during pregnancy by content experts, including a maternal–fetal medicine specialist and a master's‐level exercise physiologist trained in motivational interviewing (MI). The health coach had prior MI training through the original MyHeart trial and delivered coaching using standardized study protocols, including predefined criteria for escalation of clinical concerns to the care team. Employing an explanatory sequential mixed‐methods design, qualitative data were collected following trial completion to contextualize and expand upon the quantitative results. The study was guided by the qualitative aims and a theoretical framework integrating Self‐Determination Theory (SDT) and Self‐Regulation Theory (SRT), as illustrated in Figure 1. SDT posits that experiences of autonomy, competence, and connection with others foster motivation and engagement, helping to explain participants’ involvement in postpartum hypertension management and related health behavior change [13]. SRT emphasizes self‐monitoring and confidence in one's ability to maintain behaviors over time, providing insight into how participants sustained these changes [14]. Together, these constructs informed a deductive approach to coding and interpretation of the qualitative data.

**FIGURE 1:**
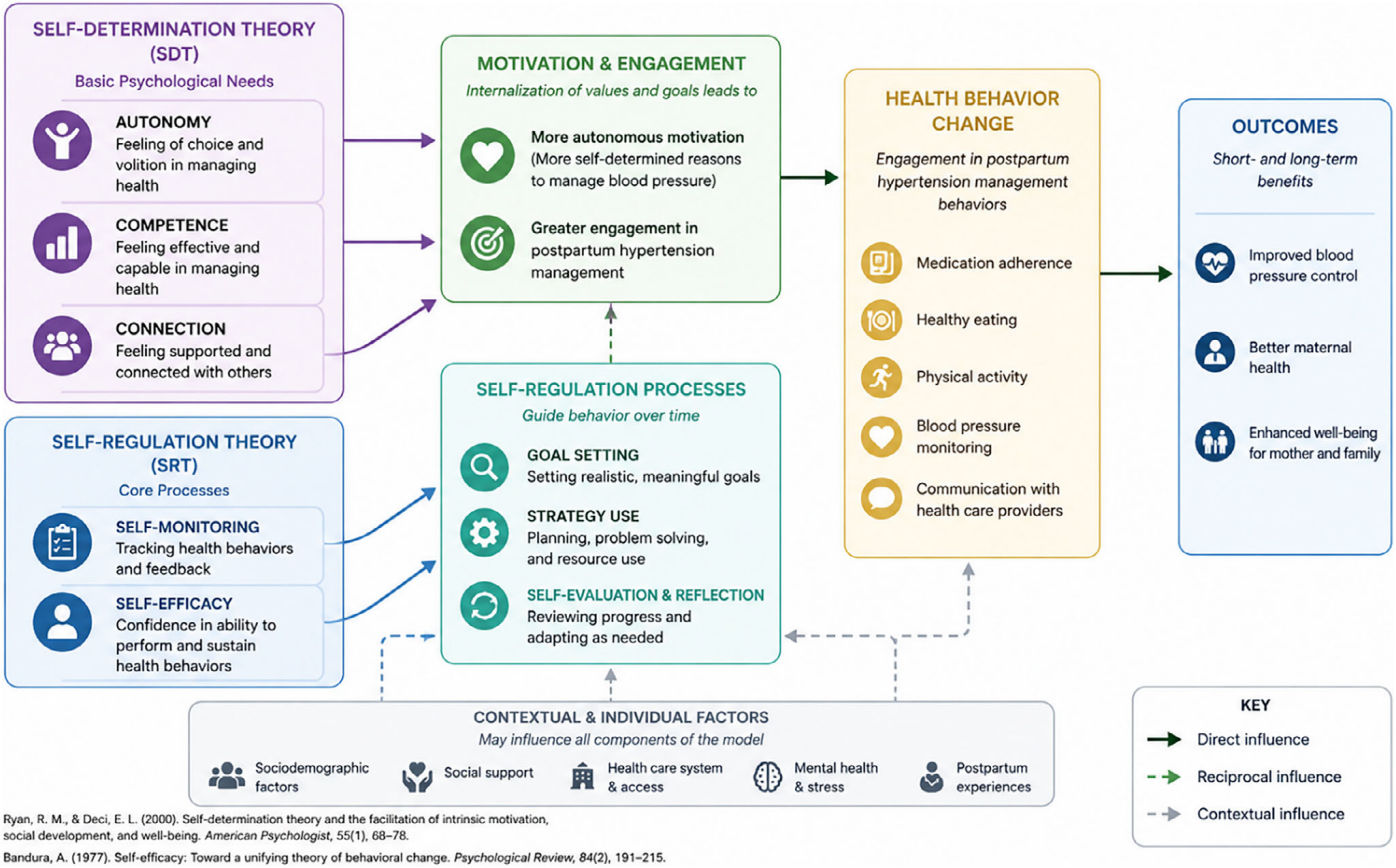
Conceptual model integrating Self‐Determination Theory (SDT) and Self‐Regulation Theory (SRT).

### 2.2 Setting and research team

The study team included five members from the Department of Obstetrics and Gynecology: a maternal‐fetal medicine specialist, a master's‐level research coordinator, a medical student, an anthropologist, and a PhD‐level public health practitioner. All interviews were conducted by the anthropologist, who had no prior relationship with participants. The research coordinator, also the intervention health coach, contributed contextual insight during analysis but did not conduct interviews. Coding was conducted in teams of two with the entire team meeting weekly to discuss coding decisions, reflexivity, and potential sources of bias, and a third coder resolved discrepancies through discussion and consensus. Study team demographics were similar to those of the participant sample.

### 2.3 Recruitment

This qualitative study was conducted as a follow‐up to a previously approved clinical trial. The study protocol was approved by the Institutional Review Board. Participants provided informed consent at enrollment into the parent trial, which included consent for participation in follow‐up qualitative interviews conducted upon completion of the trial. Participants were approached by phone for qualitative interviews, with recruitment continuing until balanced representation across study arms was achieved. Reasons for nonparticipation were tracked throughout recruitment.

### 2.4 Data collection

A semi‐structured interview guide (see the Supporting Information) was developed deductively based on trial aims and theoretical frameworks, with parallel versions for intervention and control participants, and included questions related to daily life, HDP experiences, trial participation, and self‐initiated blood pressure management. Questions about health coaching, intervention activities, and technology use were only asked of intervention participants while control participants were asked about self‐initiated behavior change and perceptions of potential coaching support. All participants were asked to provide recommendations for future health coaching interventions and postpartum care. Interviews were conducted by phone, audio‐recorded, de‐identified, encrypted, and professionally transcribed. Transcripts were reviewed for accuracy and uploaded to Dedoose for analysis. Field notes captured contextual details and reflections, which the coding team reviewed to enhance interpretation and analytic rigor.

### 2.5 Data analysis

Data were analyzed using a multi‐step thematic process. The coding team collaboratively developed a codebook, iteratively refined throughout analysis, including codes, definitions, and exemplary quotes, guided by the study aims and theoretical framework. A blended deductive–inductive approach allowed examination of both predefined constructs and emergent themes. Two coders (M.K.S. and S.G.) independently applied the codebook, documenting memos in Dedoose; a third coder (E.A.) reviewed all transcripts to resolve discrepancies. The team met regularly to refine codes, discuss reflexive memos, evaluate patterns, and generate preliminary themes. Comparative analysis across intervention and control groups identified shared experiences and group‐specific differences. A qualitative matrix with candidate themes, definitions, and quotes organized findings. Four final themes were collaboratively refined, representing patterns across both arms, with the team determining through iterative discussion that additional interviews were unlikely to yield new insights. Analysis adhered to COREQ guidelines.

## 3 RESULTS

Of the 140 trial participants, 42 were approached for qualitative interviews until 32 were completed (16 per arm). One agreed participant was unreachable; nine declined (lack of interest *n* = 6, time *n* = 2, relocation *n* = 1). Interviews occurred by phone from January 2024 to January 2025 and lasted an average of 33 min (range 15–51 min). As shown in Table 1, participants were predominantly White (87.5% in both groups), married (87.5%), reported adequate income (100% in both groups), and had a college or vocational degree or higher (82.5% in the control group and 100% in the intervention group). Hypertension type varied; chronic hypertension ranged from 37.5% in the control group to 50% in the intervention group; gestational hypertension ranged from 25% in the control group to 31.2% in the intervention group; mild preeclampsia ranged from 12.5% in the control group to 0.0% in the intervention group; and preeclampsia with severe features ranged from 25.0% in the control group to 18.8% in the intervention group. Additionally, the proportion of participants prescribed antihypertensive medication at baseline ranged from 62.5% in the control arm to 37.5% in the intervention arm.

**TABLE 1:** Participant characteristics.

Variable	Control (*n* = 16)	Intervention (*n* = 16)
Maternal		
Maternal age M (SD)	34.2 (5.2)	34.6 (3.1)
Race		
White	13 (86.7%)	14 (87.5%)
Black	0 (0.0%)	0 (0.0%)
Hispanic	1 (6.3%)	0 (0.0%)
Other	1 (6.3%)	2 (12.5%)
Missing	1 (6.3%)	0 (0.0%)
Type of insurance		
Commercial	9 (56.2%)	11 (68.8%)
HMO	5 (31.2%)	5 (31.2%)
Medicaid	2 (12.5%)	0 (0.0%)
Other	0 (0.0%)	0 (0.0%)
Unknown	0 (0.0%)	0 (0.0%)
Highest education level		
Did not finish H.S.	0 (0.0%)	0 (0.0%)
H.S. Graduate	1 (6.2%)	0 (0.0%)
Did not finish college/vocational	1 (6.2%)	0 (0.0%)
College/vocational graduate	10 (62.5%)	9 (56.2%)
At least some post‐grad school	4 (25.0%)	7 (43.8%)
Relationship status		
Single	1 (6.2%)	1 (6.2%)
Married	14 (87.5%)	14 (87.5%)
Divorced	0 (0.0%)	0 (0.0%)
Partnered	1 (6.2%)	1 (6.2%)
Self‐BP check frequency		
Not regularly	7 (43.8%)	7 (43.8%)
>1/month	3 (18.8%)	2 (12.5%)
>1/week	4 (25.0%)	3 (18.8%)
>1/day	2 (12.5%)	4 (25.0%)
Current financial status		
Difficult to pay bills	0 (0.0%)	1 (6.2%)
Cutting back on costs	0 (0.0%)	2 (12.5%)
Cannot purchase extra items	3 (18.8%)	0 (0.0%)
Can purchase extra items	13 (81.2%)	13 (81.2%)
Adequate income—yes	16 (100.0%)	13 (81.2%)
Annual household income		
No answer	0 (0.0%)	0 (0.0%)
≥$100K	10 (62.5%)	10 (62.5%)
$100K–$50K	3 (18.8%)	4 (25.0%)
$50K–$35K	1 (6.2%)	1 (6.2%)
$35K–$20K	2 (12.5%)	1 (6.2%)
<$20K	0 (0.0%)	0 (0.0%)
Obstetric		
Gravidity	2.0 (1.0–3.0)	2.0 (1.0–3.0)
Full‐term parity	1.0 (1.0–2.0)	1.0 (1.0–1.2)
Gestational age at delivery (weeks)	37.6 (1.2)	37.4 (1.3)
When pregnancy HTN diagnosed		
Prior to admit	14 (87.5%)	12 (80.0%)
Intrapartum	0 (0.0%)	0 (0.0%)
Postpartum	2 (12.5%)	3 (20.0%)
Upon readmission	0 (0.0%)	0 (0.0%)
Hypertension type		
Chronic	6 (37.5%)	8 (50.0%)
Gestational	4 (25.0%)	5 (31.2%)
Preeclampsia‐mild	2 (12.5%)	0 (0.0%)
Preeclampsia with severe features	4 (25.0%)	3 (18.8%)
Prescribed a BP medication—yes	10 (62.5%)	6 (37.5%)

Abbreviation: BP, blood pressure; HMO, health maintenance organization; HTN, hypertension.

Four final themes were identified, as illustrated along with definitions and exemplary quotes in Table 2. They capture key aspects of participants’ postpartum experiences with HDPs and engagement with health coaching, highlighting both shared experiences and differences between intervention and control groups.

**TABLE 2:** Themes from postpartum experiences with HDP and health coaching.

Theme	Description	Exemplary quotes
1. Heightened anxiety and vigilance	Participants described pregnancy, labor, delivery, and postpartum experiences as medically complex due to HDP, accompanied by persistent fear, anxiety, and hypervigilance about blood pressure and health. Both intervention and control participants noted emotional stress, uncertainty, and heightened awareness.	“Even during delivery, I was on edge. I knew the risks of my condition, and it wasn't just about the labor pain—it was about fear of what could go wrong, for me and the baby. It felt like no matter what, I had to be alert every second.” (017, Intervention) “It was exhausting…emotionally, mentally, and physically. I kept thinking about all the what‐ifs, about my blood pressure, about whether I was doing everything right.” (040, Control)
2. Desire for education and support	Participants expressed a strong need for education and guidance to manage HDP and navigate the postpartum period. Intervention participants described coaching as filling this gap, while control participants highlighted persistent informational deficits and feelings of powerlessness.	“The coaching helped me understand my numbers, what they meant day to day, and how to respond…which gave me confidence to ask questions and advocate for myself.” (033, Intervention) “I wished someone had walked me through the process, explained what to expect, or given me tools to monitor myself. I kept feeling like I had to figure it all out on my own.” (040, Control)
3. Benefits of health coaching across multiple domains	Coaching provided motivation, accountability, confidence, social and emotional support, adoption of healthy behaviors (BP monitoring, medication adherence, nutrition, hydration, physical activity), and increased engagement/self‐advocacy within the healthcare system. Control participants recognized potential benefits of coaching.	“Knowing someone would check my numbers and follow up with me made me stick to tracking my blood pressure and weighing myself. I didn't want to let the coach down.” (029, Intervention) “I didn't have coaching, but I can see how having someone check in regularly, explain things, and help me set goals would have taken a lot of stress off me.” (038, Control)
4. Unmet postpartum needs	Participants across groups described gaps in postpartum care, including limited mental health and social support, inadequate paid leave, barriers to accessing care, and insufficient attention to maternal health. Coaching partially mitigated these gaps but did not fully address them.	“After my six‐week check, it felt like everyone disappeared. I was left to deal with my blood pressure, recovery, and emotional stress on my own.” (046, Control) Having someone to talk through my worries made a huge difference, but there's no substitute for a system that routinely checks on mental health and emotional wellbeing.” (017, Intervention)

Abbreviations: BP, blood pressure; HDP, hypertensive disorder of pregnancy.

### 3.1 Theme 1: Hypertensive disorders of pregnancy heightens fear and anxiety

Participants described their pregnancy, labor, delivery, and postpartum period as medically complex due to HDP. In addition to these medical challenges, many reported intense anxiety, persistent fear, and heightened vigilance about their blood pressure, which permeated daily life. One participant (033, Intervention) shared, “Every [perinatal healthcare] appointment felt like walking a tightrope. I was constantly worried about whether my blood pressure would spike, whether I would need to be hospitalized again, or whether something could go wrong for the baby. It made me hyperaware of every symptom, and sometimes it was overwhelming to process everything alone.” Similarly, another intervention participant (017, Intervention) described the emotional weight of managing complications: “Even during delivery, I was on edge. I knew the risks of my condition, and it wasn't just about the labor pain—it was about fear of what could go wrong, for me and the baby. It felt like no matter what, I had to be alert every second.”

Control participants echoed these experiences, emphasizing the stress and uncertainty associated with their conditions in the absence of structured support. One control participant (046) reflected, “I felt like I was navigating this high‐risk pregnancy blindfolded. Every symptom made me anxious, and I wasn't sure who to call or what to do. After delivery, I just felt completely dropped off. I didn't feel like anyone was looking out for me.” Another control participant (040) described similar emotional strain: “It was exhausting…emotionally, mentally, and physically. I kept thinking about all the what‐ifs, about my blood pressure, about whether I was doing everything right. It wasn't just labor or delivery…it was the constant worry that never went away.”

### 3.2 Theme 2: Desire for hypertension disorders of pregnancy education and support

Participants expressed a strong desire for HDP‐related education and support to help them manage hypertensive disorders and navigate the postpartum period. Many expressed that understanding the condition better would have empowered them to advocate for themselves and feel more in control. Control participants frequently noted gaps in information from their providers. One control participant (046, Control) shared, “I wanted to understand what my blood pressure numbers really meant, what was normal, what was dangerous, and what I could do about it. I kept asking questions, but I never got a clear answer. It left me feeling powerless.” Similarly, another control participant (040) reflected, “I wished someone had walked me through the process, explained what to expect, or given me tools to monitor myself. I kept feeling like I had to figure it all out on my own.”

Intervention participants echoed this need for education and support but described how the health coaching intervention helped fill the gap. Participant 033 (Intervention) noted, “The coaching helped me understand my numbers, what they meant day to day, and how to respond. It wasn't just being told what to do…it was knowing why, which gave me confidence to ask questions and advocate for myself.” Another intervention participant (017, Intervention) highlighted the empowerment gained: “I could ask about specific symptoms or patterns, and my coach explained what was normal, what was risky, and how I could act. I felt like I had a safety net of knowledge that I didn't have before.”

Across both groups, participants expressed that knowledge, education, and support are essential to reduce anxiety, increase confidence, and promote engagement with healthcare providers. The intervention participants’ experiences suggest that structured support, like health coaching, can fulfill these desires and enhance empowerment.

### 3.3 Theme 3: Benefits of health coaching across multiple domains

Intervention participants described multiple benefits of health coaching, including increased motivation and confidence, social support, lifestyle changes, and greater engagement and self‐advocacy within the healthcare system. Intervention participants consistently reported that coaching provided structure, motivation, and reassurance in ways that standard care did not. Coaching helped them set and stay on track with goals, maintain accountability, and build confidence in managing their health and blood pressure. Participant 029 (Intervention) explained, “Knowing someone would check my numbers and follow up with me made me stick to tracking my blood pressure and weighing myself. I didn't want to let the coach down, and it made me more consistent than I would have been on my own.” Participant 017 (Intervention) added, “It wasn't nagging…it was encouragement. Having someone care about whether I followed through really motivated me.”

Participants valued the emotional encouragement and sense of connection offered through coaching. Regular check‐ins and personalized attention helped reduce fear and anxiety, making participants feel supported and understood during the postpartum period. Participant 033 (Intervention) shared, “Even when my blood pressure spiked, I felt calmer because I knew I could talk through it with someone who understood, who wouldn't judge, and who gave me clear guidance.”

Health coaching supported participants in adopting and sustaining concrete health behaviors, including consistent blood pressure monitoring, adherence to medication, increased physical activity, and improved nutrition and hydration. Participants described how goal‐setting, guidance, and accountability made these changes more achievable and manageable within their daily lives. Participant 029 (Intervention) explained, “She helped me break things into small, achievable goals—like walking daily and prepping meals in advance. It didn't feel overwhelming, and I actually did it.”

Participants reported increased confidence in communicating with healthcare providers and advocating for their own care. Coaching helped them ask questions, seek necessary follow‐up, and navigate the healthcare system more effectively, strengthening their role in managing postpartum hypertension. Participant 033 (Intervention) noted, “I felt more confident calling the doctor when something felt off because I knew my coach had my back. I wasn't just passively going along; I could ask questions, clarify instructions, and advocate for myself.” Participant 011 (Intervention) reflected, “Having that support made me less intimidated to speak up in [perinatal healthcare] appointments. I felt like I had a partner in navigating the system.”

Even though they didn't receive the intervention, control participants also recognized the potential benefits of health coaching. Participant 038 (Control) shared, “I didn't have coaching, but I can see how having someone check in regularly, explain things, and help me set goals would have taken a lot of stress off me.” Another control participant (046, Control) said, “Just knowing someone was keeping an eye on things and helping me stay on track would have been invaluable. It would have reduced my anxiety and helped me feel less alone.”

Overall, health coaching was perceived as valuable not only for managing hypertension but also for supporting emotional well‐being, lifestyle behaviors, and engagement with the healthcare system. Control participants highlighted that structured coaching could have provided similar benefits, underscoring the intervention's perceived importance.

### 3.4 Theme 4: Unmet needs in the postpartum period

Participants from both groups described significant gaps in postpartum care, emphasizing the need for consistent, holistic support throughout the postpartum period and transition into ongoing care. Key gaps included limited mental health and social support, inadequate paid leave, barriers to accessing care, and insufficient attention to overall maternal health. Control participants often reported feeling abandoned after delivery, experiencing both physical and emotional challenges in isolation. Participant 046 (Control) explained, “After my six‐week check, it felt like everyone disappeared. I was left to deal with my blood pressure, recovery, and emotional stress on my own. It was terrifying and lonely.” Participant 040 (Control) described similar feelings, “It was hard not having someone to guide me. I felt anxious, exhausted, and unprepared for what came next.”

Intervention participants emphasized that health coaching partially mitigated these gaps by providing ongoing support, yet they also recognized that broader, comprehensive postpartum care is needed. Participant 033 (Intervention) said, “The coaching helped me feel checked on and cared for, but I still see how much more comprehensive care is needed—screening for depression, anxiety, and lifestyle support. One‐on‐one coaching is helpful, but the system as a whole needs to step up.”

Mental health concerns were prominent in this theme. Participants reported postpartum stress, anxiety, and depressive symptoms, often exacerbated by gaps in care. Participant 017 (Intervention) reflected, “Having someone to talk through my worries made a huge difference, but there's no substitute for a system that routinely checks on mental health and emotional wellbeing.” Control participants similarly noted that emotional support was largely absent, contributing to feelings of isolation and uncertainty.

Across groups, participants voiced a clear desire for comprehensive, continuous postpartum care that integrates blood pressure monitoring, mental health support, lifestyle guidance, and clear education. Participants emphasized that addressing both medical and emotional needs in a coordinated manner could prevent feelings of abandonment and improve health outcomes for birthing people with and without HDP. One participant emphasized, “They always care about the baby…you kind of forget about the mom. We need to keep track of her too.”

## 4 DISCUSSION

Our findings highlight the postpartum experiences of individuals with HDP and the perceived value of postpartum health coaching. Participants described pregnancy, labor, and delivery as emotionally complex, marked by fear, anxiety, and heightened vigilance. Across both groups, participants emphasized a strong need for education and support to navigate these challenges, while intervention participants described how health coaching helped fill this gap.

Engagement in coaching was associated with benefits spanning motivation, accountability, confidence in self‐management, lifestyle changes, and greater involvement and self‐advocacy within the healthcare system. These benefits aligned with core constructs of SDT, particularly autonomy and competence, as well as SRT, including self‐monitoring and self‐efficacy. Experiences reflecting SDT‐related relatedness—especially through peer or broader community connections—were less evident and may represent an important area for future research. Notably, participants in the control group also acknowledged the potential value of structured support, underscoring both shared needs and meaningful differences across study groups.

These findings not only align with prior research showing that health coaching can increase awareness of cardiovascular risk and promote lifestyle changes [15, 16], but also reveal additional psychosocial benefits, such as emotional support and increased self‐advocacy, that have received comparatively little attention in previous studies. Comparison across intervention and control groups underscored that while both groups valued education and guidance, coaching actively facilitated goal setting, accountability, and confidence in ways standard care did not.

Despite growing use of health coaching in postpartum care, there is currently no standardized set of components for individuals with HDP. While features such as goal setting, motivational interviewing, and connection to healthcare providers are linked with positive outcomes [17], a unified certification pathway to implement more standardized, evidence‐informed coaching in postpartum hypertension care has not been established [18]. Expanding the pool of providers who can deliver behavior change support, including community health workers, doulas, nurses, and exercise physiologists, may offer meaningful benefits, particularly in communities underserved by traditional healthcare systems.

The American College of Obstetricians & Gynecologists (ACOG) recommends early and frequent postpartum blood pressure follow‐up for individuals with HDPs, including assessment within 72 h to 7–10 days postpartum [4]. This study demonstrates that individuals with HDP face persistent gaps in postpartum care, including limited education, support, and coordinated services. Health coaching offers a promising strategy to address these gaps by providing structured guidance, emotional support, and tools for self‐management and advocacy. Integrating evidence‐informed coaching into postpartum care could enhance both patient experience and long‐term outcomes for this high‐risk population.

### 4.1 Strengths and limitations

This study has several strengths, including the inclusion of both intervention and control participants, which allowed for the identification of unique experiences and perceptions attributable to health coaching. The study centered participants’ experiences within the broader postpartum context and employed a rigorous, iterative, and collaborative qualitative analytic process, guided by theoretical frameworks.

Limitations include the largely homogenous sample, which was predominantly White, married, high income, and highly educated. The perspectives of individuals from other racial and ethnic groups, lower‐income backgrounds, and Medicaid beneficiaries were underrepresented. Considering this, future research should intentionally overrecruit underrepresented groups, specifically populations disproportionately affected by the HDP, to better understand experiences in health coaching and HDP follow‐up postpartum care to ensure generalizability and cultural relevance of findings.

### 4.2 Implications

Structured health coaching can serve as a valuable complement to routine postpartum care for individuals with HDP by providing ongoing monitoring, personalized education, emotional support, and guidance for lifestyle change. Integrating coaching into clinical workflows—or through nontraditional providers such as nurses, community health workers, or doulas—could enhance engagement, self‐management, and maternal well‐being beyond the standard 6‐week postpartum visit. Developing training programs and implementation protocols is essential to ensure consistent, effective delivery.

These findings highlight the need for policies that expand access to comprehensive postpartum programs, including coverage for structured coaching services. Establishing reimbursement frameworks could promote equitable, sustained support for high‐risk populations. Standardization of coaching models and integration into maternal health policies are critical for scaling interventions and reducing disparities. Currently, health coaching is a billable service in some settings [18], but standardization and reimbursement remain limited, highlighting the need for policy frameworks that support equitable, sustained postpartum care.

Future research should examine the effectiveness, scalability, and equity of health coaching interventions for diverse postpartum populations, particularly Black birthing people, who experience maternal mortality rates more than twice that of White individuals and a fivefold higher risk of death from preeclampsia or eclampsia [19], and those with Medicaid or low‐income status. Studies should explore optimal coaching delivery models, integration with HBPM, and long‐term impacts on cardiovascular outcomes, mental health, and healthcare engagement. Additionally, understanding how coaching can address gaps in social support and peer/community connection may inform the development of more holistic, patient‐centered interventions.

## 5 CONCLUSION

Individuals with HDPs face significant emotional, informational, and healthcare challenges in the 1‐year postpartum period, including fear, isolation, and uncertainty. This study demonstrates that structured health coaching can meaningfully address these challenges by fostering motivation, confidence, accountability, and self‐advocacy, while supporting lifestyle change and engagement with the healthcare system. However, coaching alone does not fully bridge persistent gaps in mental health support, education, and continuity of care. Integrating evidence‐informed coaching into comprehensive postpartum care offers a promising strategy to enhance both patient experience and long‐term cardiovascular and psychosocial outcomes. Centering patient perspectives in intervention design and implementation is essential to developing equitable, responsive, and scalable postpartum care models for this high‐risk population.

## AUTHOR CONTRIBUTIONS

**Kristine Buchholz**: Writing—original draft; formal analysis. **Elizabeth Albert**: Conceptualization; writing—review and editing; formal analysis; methodology. **Kara K. Hoppe**: Conceptualization; writing—review and editing; supervision. **Megan R. Knutson Sinaise**: Writing—review and editing; formal analysis. **Melissa Neal**: Writing—review and editing; formal analysis. **Scott Hetzel**: Formal analysis. **Srishti Gupta**: Writing—review and editing.

## CONFLICT OF INTEREST STATEMENT

The authors declare no conflicts of interest.

## Supporting information



Supporting information
