# Supplementary material for: Bridging care gaps: A qualitative study of postpartum health coaching for hypertensive disorders of pregnancy
Source: Pregnancy (Hoboken). 2026 Jul 10;2(4):e70354. doi: 10.1002/pmf2.70354 (PMC13624838; doi:10.1002/pmf2.70354)
Supplement: Supplementary file 1 [file PMF2-2-e70354-s001.docx]

**Supplemental Material: STAC-MyHEARTp Exit Interview Guide**

Participants were recruited from both the intervention and control arms of the STAC-MyHEARTp study and completed interviews approximately 12 months postpartum. Interviews were semi-structured and interviewers used prompts, probes, and follow-up questions as appropriate.

**Introduction and Consent**

Prior to turning on the recorder, interviewers introduced themselves, asked the participant’s name, addressed participant questions, and obtained verbal informed consent.

Thank you for taking the time to speak with me today. I’m interested in learning about your experiences with hypertension in pregnancy and how you felt participating in the STAC-MyHEARTp study. Hearing your story in your own words will help us better understand the experiences of people with high blood pressure in pregnancy and improve future versions of this study. There are no right or wrong answers, and you may skip any question or stop the interview at any time.

**Part 1: Intro Questions Asked of All Participants**

**Participant Background**

- Where did you grow up?
- Where do you live now? Who else lives with you, if anyone?
- Tell me about what keeps you busy during the day.
- If employed or in school, what do you study or do for a living?

**Birth and Postpartum Experience**

- Was this your first birth?
  - Prompt: How long did you labor?
  - Prompt: When did you decide to go to the hospital?
  - Prompt: Who was there with you?
  - Prompt: Did you have any kind of doula support?
- Did you create a birth plan or have specific ideas about how you wanted your birth experience to happen?
  - If yes:
    - Did your labor and birth happen how you expected?
    - How are you feeling/doing?
    - How is baby feeling/doing?
  - If no:
    - How was your experience?
    - How did that make you feel?
    - How are you doing?
    - How is baby feeling/doing?
- How has your postpartum period been?
- Have you felt supported by your healthcare team?
- How have you been feeding your baby (breastfeeding, pumping, formula)?
- How long did you breastfeed or pump?
  - Prompt: How was that experience for you?
- When did you decide to introduce formula?

**Hypertension and Cardiovascular Risk**

- Was this your first pregnancy?
- If not, did you experience hypertension in prior pregnancies?
- Does your family have any history of high blood pressure or chronic hypertension?
  - Prompt: Was hypertension or preeclampsia something you were aware of prior to becoming pregnant?
- At the time you were recruited into the study, were you aware that hypertension in pregnancy could increase future cardiovascular disease risk?
  - If no:
    - When did you realize this?
    - How did that make you feel?
    - Prompt: How did it feel to hear your blood pressure was still high 6-weeks postpartum?
  - If yes:
    - Where did you learn about that cardiovascular risk?
    - What was going through your mind at that time?
    - Prompt: How did it feel to hear your blood pressure was still high 6-weeks postpartum?
- Were you placed on blood pressure medication?
  - Prompt: How did that make you feel?
  - If breastfeeding or pumping:
    - Were you worried about medication exposure?
- Did you experience any medication adjustments?
  - If yes:
    - How did that make you feel?
    - Do you think being in the study gave you greater access to care?
    - What do you think you would have done if you were not in the study?
  - If no:
    - How did that make you feel?
    - What were you doing to maintain your blood pressure without medication adjustment?

**Recruitment and Retention**

- Why did you decide to participate in this study?
  - Prompt: What was your motivation?
- What difficulties or hardships did you experience in the study?
- Was there any point when you thought about dropping out?
  - Prompt: Why?
  - Prompt: What was going on at that time?
  - Prompt: What motivated you to remain in the study?

**Part 2: Questions Asked Only of Intervention Arm Participants**

**Study Experience**

- How did you feel being placed (randomized) into the intervention group?
- How was the experience of engaging in the different study activities?
  - Prompt: Was participation overwhelming?
  - Prompt: Did activities feel like too much or a waste of time?
- What do you think it would have been like without the health coaching, blood pressure monitor, or weight scale?

**Blood Pressure Monitoring**

- How did you feel monitoring your blood pressure every week?
  - Prompt: Was it helpful?
  - Prompt: Did it cause anxiety?

**Weight Monitoring**

- How did you feel using the weight scale?
  - Prompt: Was it helpful?
  - Prompt: Did it cause anxiety?

**Health Coaching and Education**

- What did you like about receiving health coaching?
- What did you not like about receiving health coaching?
- How did you feel having someone help manage your health goals?
- What did you learn?
- Did you seek information outside of the study?
  - Prompt: Where?
- Did you make or sustain behavior or lifestyle changes?
  - Prompt: Have you noticed changes in your blood pressure because of those changes?
  - Prompt: How will you continue these changes now that health coaching is over?

**Technology**

- What was your experience with the technology?
  - Prompt: Blood pressure monitor.
  - Prompt: Weight scale.
  - Prompt: Is there anything we could have done better?

**Future Participation**

- If continued health coaching was offered after the study ended, would you be interested?
- What type of health coaching would you be most interested in?
  - Prompt: Exercise or personal training.
  - Prompt: Nutrition.
  - Prompt: Heart health.
  - Prompt: Mental health.
- For how long would you want health coaching?
  - Prompt: Six months.
  - Prompt: One year.
  - Prompt: Longer.
- At what intervals would you prefer contact?
  - Prompt: Monthly.
  - Prompt: Every three months.
- If you became pregnant again, would you want to continue or restart health coaching?
  - Prompt: Why would that be important to you?
- Would you be willing to participate in future postpartum follow-up visits involving cardiac testing?
  - Prompt: Ultrasound of the heart.
  - Prompt: Stress test.
- Would you be willing to complete future surveys or interviews at 1, 2, or 5 years postpartum?
  - Prompt: We would be interested in how health changes were maintained over time.

**Part 3: Questions Asked Only of Control Arm Participants**

**Study Experience**

- How did you feel being placed (randomized) into the control arm?
- What do you think it would have been like to have access to a weight scale or health coaching through 12 months postpartum?

**Blood Pressure Monitor Use**

- Did you receive the blood pressure monitor when you enrolled?
- Did you use it prior to your final call with the health coach?
  - If yes:
    - How often did you use it?
    - Was it helpful?
      - Prompt: In what ways?
    - What did you do with the results?
    - Did you share the results with a healthcare provider or anyone else?
  - If no:
    - Why not?
    - What did you do with the monitor in the meantime?

**Behavior Change and Information Seeking**

- Did you do anything to create or sustain changes to help manage your blood pressure?
- What changes did you make or attempt to make?
- Where were you finding information?
- Did you spend money on services or information related to blood pressure management?
  - Prompt: How much?

**Future Participation**

- If health coaching was offered after the study ended, would you be interested?
- What type of health coaching would you be most interested in?
  - Prompt: Exercise or personal training.
  - Prompt: Nutrition.
  - Prompt: Heart health.
  - Prompt: Mental health.
- For how long would you want health coaching?
  - Prompt: Six months.
  - Prompt: One year.
- At what intervals would you prefer contact?
  - Prompt: Monthly.
  - Prompt: Every three months.
- If you became pregnant again, would you want to participate in health coaching?
  - Prompt: Why would that be important to you?
- Would you be willing to participate in future postpartum follow-up visits involving cardiac testing?
  - Prompt: Ultrasound of the heart.
  - Prompt: Stress test.
- Would you be willing to complete future surveys or interviews at 1, 2, or 5 years postpartum?
  - Prompt: We would be interested in how health changes were maintained over time.

**Part 4: Closing Questions Asked of All Participants**

**Overall Recommendations**

- When we do this project again, what are the most important things we could do to improve the study?
- Do you have recommendations for improving postpartum care?

**Closing**

- In thinking about your experience in the study, can you summarize in a sentence or two what this has been like for you?
- Is there anything we did not discuss about your experience with hypertension or participation in the study that you would like to share?
  - Prompt: Repeat until participant has nothing further to add.
- Is there a particular pseudonym you would like us to use for you, or should we choose one?
